# A Re-evaluation of Online Pornography Use in Germany: A Combination of Web Tracking and Survey Data Analysis

**DOI:** 10.1007/s10508-023-02666-8

**Published:** 2023-08-29

**Authors:** Maximilian T. P. von Andrian-Werburg, Pascal Siegers, Johannes Breuer

**Affiliations:** 1 Institute Human-Computer-Media, Faculty of Human Sciences, University of Wuerzburg, Oswald-Kuelpe-Weg 82, 97074 Wurzburg, Germany; 2 GESIS–Leibnitz Institute for the Social Sciences, Mannheim, Germany; 3 Center for Advanced Internet Studies, Bochum, Germany

**Keywords:** Online pornography use, Web tracking data, Religiosity, Sexism, Social dominance orientation

## Abstract

Several researchers have questioned the reliability of pornography research's findings. Following a recent call to use more reliable data sources, we conducted two analyses to investigate patterns and predictors of online pornography use (OPU). Our analyses were based on data from a large-scale German online web tracking panel (*N* = 3018) gathered from June 2018 to June 2019. The study we present here has two parts: In the first part, we looked at group differences (gender and age) in tracked OPU. Overall, this part's results confirm questionnaire-based research findings regarding sex and age differences. In the second part of our study, we combined the web tracking data with data from an online survey which was answered by a subset of the tracking participants (*n* = 1315) to assess the relevance of various predictors of OPU that have been identified in previous research. Again, our results mostly echoed previous findings based on self-reports. Online pornography was used more by males and younger individuals, while relationship status, sexist attitudes, and social dominance orientation were not associated with OPU. However, we did find differences in OPU between members of different religious communities. Our study confirms some critical findings on OPU from previous questionnaire-based research while extending existing research by providing a more fine-grained analysis of usage patterns based on web tracking data.

**Supplementary Information:**

The online version contains supplementary material available at 10.1007/s10508-023-02666-8.

## Introduction

For reasons deeply rooted within the phylogeny, humans are by themselves prone to externalize memory, thoughts, and fantasies, including those of a sexual nature (von Andrian-Werburg et al., 2023). According to a standard definition, “Pornography refers to any type of sexually explicit material that has the intent of producing arousal in those who consume it” (Lehmiller, 2017, p. 402). While the reasons for people to use pornography (e.g., Burtăverde et al., 2021) probably have stayed the same across human history, the ways have changed how pornography is produced, distributed, and consumed. From the printing press to television and video to the internet, there has been a steady increase in the availability and accessibility of pornographic content. Today, for most people across the globe, the internet has become the main venue for the consumption of pornography (see, e.g., Grubbs et al., 2022; Salmon et al., 2020). Research suggests that the spread of high-speed internet connections is associated with increased pornography use (e.g., Lewczuk et al., 2022). According to data from the US General Social Survey (Price et al., 2016), for instance, the relative share of users within the population of young men aged 18–26 rose from 51.8 to 61.1% between 1998 and 2007, which marks an increase of 9.3% points. For young women of the same age, a 6.9% point increase, from 29.2 to 36.1%, occurred within the same time frame (Price et al., 2016, p. 5). Similar but smaller trends could also be found for other age cohorts in the US.

There is a rich body of research on pornography use (PU) in general and online pornography use (OPU) in particular, as well as their potential predictors and outcomes. The predominant method to study these topics—at least in fields within or related to the quantitative behavioral and social sciences—have been surveys. Surveys allow for an economic assessment of online and offline PU and associated psychological and behavioral phenomena. As such, survey-based research has produced valuable insights into PU's antecedents, types, and consequences. However, surveys are typically a type of self-report, and these self-reports can have limitations, especially when studying PU.

Notably, the quality of questionnaire responses depends on participants' will and ability to disclose “true” thoughts, behavior, and feelings (e.g., Rasmussen et al., 2018). Hence, the method is vulnerable to biases, such as an underreporting of PU based on differences in individual perceptions of which media content could be described as pornographic, recall bias, or social desirability (Kohut et al., 2020; Morichetta et al., 2021; Rasmussen et al., 2018). Supporting this assumption, using passively tracked web traffic data, Morichetta et al. (2021) found an average OPU of 37 min per week compared to 24 min reported in a comparable survey-based study. This discrepancy aligns with a systematic review and meta-analysis that finds discrepancies between logged and self-reported digital media use in general (Parry et al., 2021).

Furthermore, questionnaire-based pornography research often suffers from limitations not inherently attached to the questionnaire method but issues related to the respective methodological implementation (Kohut et al., 2020; Marshall & Miller, 2019). Many studies do not work with a clear operational definition of pornography and PU despite previous work that provides such definitions (Kohut et al., 2020; Lehmiller, 2017; Marshall & Miller, 2019). This ambiguity in construct operationalization has resulted in the problematic use of different scales and scale anchors to assess PU (Kohut et al., 2020; Marshall & Miller, 2019). This lack of corresponding operationalizations, in turn, can make it difficult to compare (the results of) individual studies in the field. Another area for improvement of many studies on PU is that they are based on cross-sectional data, which does not allow for the analysis of longer-time trends. The relatively few existing longitudinal studies typically only include a small number of survey waves (Grubbs et al., 2022; Martyniuk & Dekker, 2018; Price et al., 2016). Because of these limitations of existing PU research, Grubbs and Kraus (2021) have recently called for replicating and extending existing questionnaire-based findings with alternative/additional data types. Web tracking data is one type that might be suitable for a response to the call.

So far, only a few studies on (O)PU have used web tracking. The statistics blog Pornhub Insights frequently publishes usage trends based on the web traffic on Pornhub (e.g., Pornhub Insights, 2021). However, these are not peer-reviewed scientific publications, are limited to one website, and are based on an undisclosed database. In a pioneering academic study, Morichetta et al. (2021) analyzed the anonymized traffic of about 15,000 broadband subscribers between March 2014 and 2017 regarding patterns and types of OPU. Among other things, the study found a sharp increase in smartphone use for accessing online pornography (OP) during the assessed time frame. The authors also found temporal trends of OPU, which rises when more leisure time is available. Furthermore, the study demonstrated that only a few websites are responsible for the leading share of OP traffic. In another study based on web tracking data, Lewczuk et al. (2022) discovered a sharp increase in OPU among Polish internet users, with the number of OP users tripling from an estimated 2.76 million in 2004 to 8.54 million in 2016.

Our study aims to extend survey-based (O)PU research and the recent pioneering work based on web tracking data. As web tracking data alone are also limited in several regards—most notably a lack of information about the individual users—we used a combination of web tracking and survey data to use the unique strengths of both data types (Stier et al., 2020). Our study consists of two parts:

In the first part, we present an exploratory analysis to assess the replicability of descriptive survey-based findings on OPU. In the second part, we present a theory-guided correlational analysis of different (types) of predictors of OPU. More specifically, based on existing studies that have identified critical correlates of (O)PU, we look at the role of sex and age (e.g., Martyniuk & Dekker, 2018; Price et al., 2016), relationship status (Morgan, 2011), religiosity (Grubbs et al., 2019; Short et al., 2015; Willoughby & Busby, 2016; Štulhofer et al., 2022), as well as (possibly) relevant attitudinal dimensions, such as sexism (Kohut et al., 2016; Speed et al., 2021). Again, the fundamental research interest is to assess to what degree survey-based results can be replicated with web tracking data combined with survey data.

## Part One: Descriptive Analysis to Replicate Survey-Based Research on Pornography Use Patterns With Web Tracking Data

While, as discussed above, some patterns of PU have changed due to the rise of high-speed internet connections, one pattern consistently found across time and cultures (e.g., Hald & Mulya, 2013) is that men use far more pornography than women (Martyniuk & Dekker, 2018; Price et al., 2016). Another pattern typically found is that there are age differences in PU. For example, in the US General Social Survey, the cohort with the highest share of pornography users is the group of 18-to-26-year-olds (Price et al., 2016). In older cohorts, the relative share of pornography users (as reported in survey studies) declines. The picture for Germany is slightly different, according to the representative survey study by Martyniuk and Dekker (2018). Here, the cohort with the largest relative share of pornography users is the 31-to-45-year-olds, where 90% of men and 42% of women reported using pornography. As our data originate from Germany, the survey study by Martyniuk and Dekker (2018) is the main reference point for the first part of our study. The overall research question for this part of our study is: Can findings regarding sex and age differences in OPU based on self-reported data be replicated with web tracking data?

### Method

To answer our research question in Part One, we used web tracking data from a multipurpose study that originally aimed not on pornography research. The web tracking data stems from a non-probability panel of German Internet users that a German market research company manages following the ICC/ESOMAR International Code of Marketing and Social Research Practice (see https://iccwbo.org/publication/iccesomar-international-code-on-market-and-social-research/). Participants in the panel consented to install and use a browser extension that tracks their web browsing behavior on desktop computers and mobile devices (depending on what they opted for). Participants received monthly monetary incentives for tracking from the market research company. For privacy reasons, the tool includes the possibility to pause the tracking. The data we used for our study spans from June 2018 to June 2019 and includes information about website visits on the domain level (e.g., Pornhub.com). In addition to the domain and the time of the website visit, the tracking tool recorded its duration (in seconds). The web tracking panel includes around 2000 participants per month. The sample size fluctuates a bit due to participants dropping out and new ones being recruited into the panel to fill in for the participants who had dropped out. In addition to the web tracking data, the company that offers the panel also provided us with basic demographic information about the participants (such as age and sex). For the full-time period, our sample contains *N* = 3018 unique participants (women = 1537, men = 1481) aged between 18 and 65 (*M*_age_ = 41.9 years, *SD*_age_ = 13.46 years).

The web tracking data also include automatically generated classifications of the websites. The category of interest for our analyses was labeled “adult.” However, as this category also contained websites that cannot be identified as OP (e.g., dating platforms focused on casual sex), we manually categorized the 284 most popular adult websites with more than 1000 visits in our sample into different structural categories. We only included visits to pornographic video, photo, and camera portals in our analyses in our study, resulting in a total of 214 domains from our coded list. Accordingly, OPU in our study is operationalized as visits to an online pornographic video, photo, or live camera portal. Our data set includes a total of 2,020,226 such website visits from 1386 unique OP users (29% female). In the raw data, each call to a new URL (within the same domain) counts as a visit. As the duration is measured in seconds, the shortest possible duration for a visit is one second. For our analyses, we aggregated this raw visit counts into OPU sessions following the approach used by Morichetta et al. (2021). First, we reduced the tracking data to visits to OP sites from the categories we focused on. Using this focused data set, we counted visits as belonging to a new session if there was a temporal gap of 30 min or more between said visit and the previous OP visit. Thus, if the time between individual OP visits (within the same domain or across domains) was shorter than 30 min, we counted the visits as belonging to the same OPU session.

### Results

Overall, 45.92% (*n* = 1386) of individuals in our web tracking sample (*N* = 3018) could be identified as OP users, meaning that they had at least one visit to one of the websites from the categories described in the previous section throughout our study. Among those classified as users, the average number of sessions per month was 6.4 (*SD* = 11.38).

In line with previous research, we find significant differences between men and women in OPU. While 66% of male participants in our sample were OP users, this share was much lower in our study, with 26% for women. Likewise, among the users, the average number of monthly sessions for men was 8.07 compared to 2.33 for women.

**Fig. 1:**
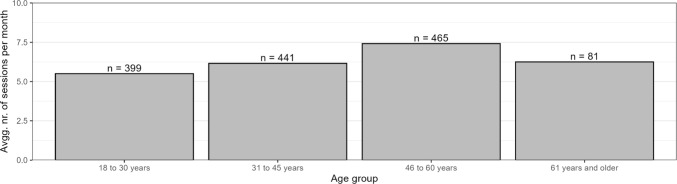
Average number of monthly online pornography use sessions per age category. *Note.* The analysis visualized here is restricted to OP users

We used the same age categories as Martyniuk and Dekker (2018) to investigate age differences in our sample. Overall, age differences in OPU are less pronounced than sex differences, but our data shows a decline in usage with age. With 52%, we have the highest share of OP users in the age group from 18 to 30, compared to 49% among participants aged 31–45, 42% for the 46-to-60-year-olds, and 33% among individuals aged 61 and above. Regarding average monthly OPU sessions, there is a curvilinear relationship with age in our data (see Fig. 1). The highest number of average OP use sessions per month is in the group of participants aged 46 to 60.

**Fig. 2:**
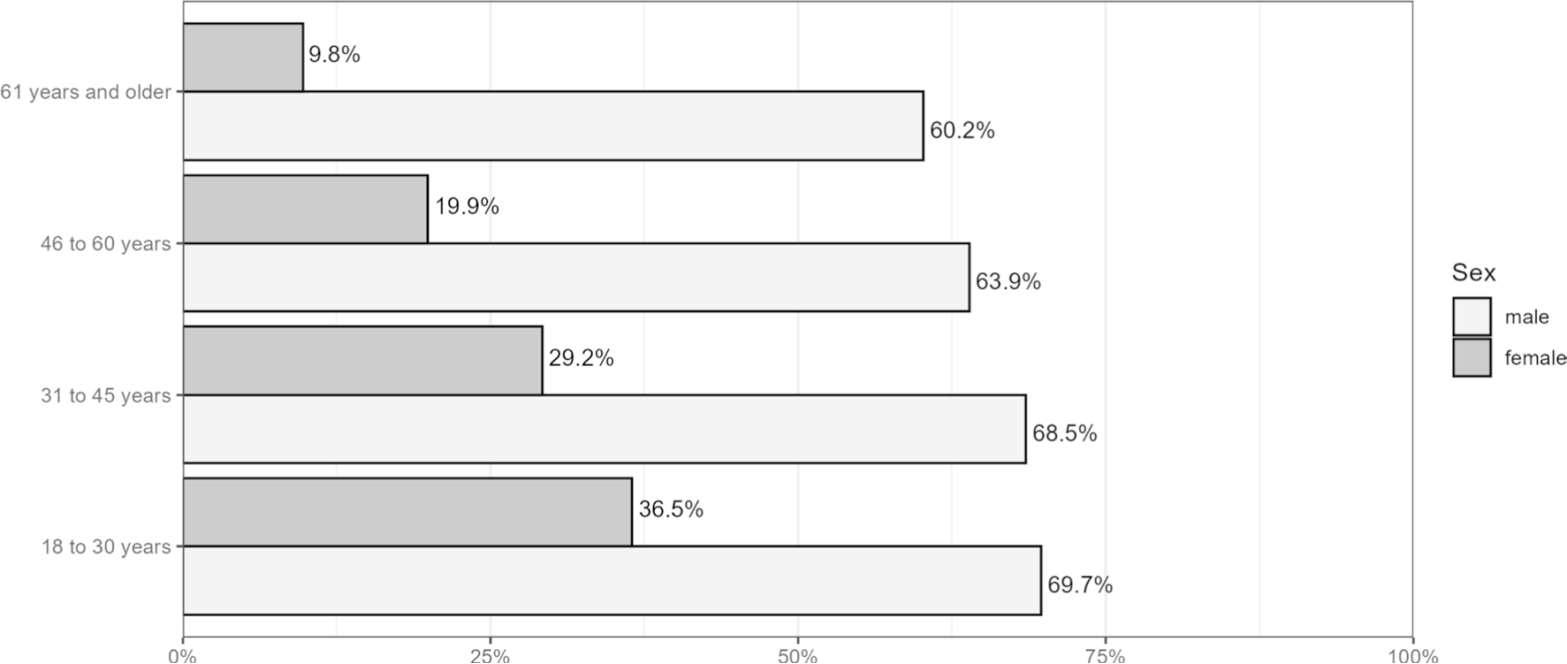
Share of online pornography users (participants with at least one porn website visit)

Following the analysis by Martyniuk and Dekker (2018), we also looked at sex differences across age groups. Replicating what they found with self-reported data, we see that sex differences of OP users increase with age (see Fig. 2). While 36.5% of women and 69.7% of men in the age group 18 to 30 engage in OPU in our sample (difference of 33.2% points), it is 60.2% for men and 9.8% for women among those aged 61 and older (difference of 50.4% points). This illustrates that the decline in user shares with age is much smaller for men than women.

**Fig. 3:**
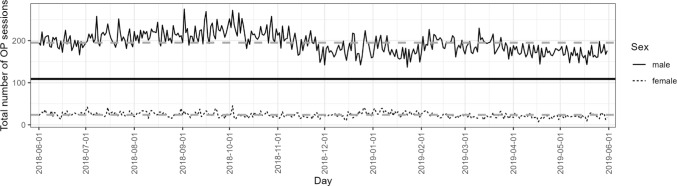
Total number of online pornography use sessions per day separate for each sex*. Note.* The solid straight line represents the overall mean, and the dashed straight lines represent group means

To complement our group comparisons on the aggregate level, we also looked at the total daily sessions across our observational period by sex (see Fig. 3). The number of daily sessions for female users is consistently lower and shows much less variation across time.

### Discussion

Regarding our research question, overall, we could reproduce the key findings on sex and age differences in OPU from the self-report study by Martyniuk and Dekker (2018) with our web tracking data. Despite the reasonable criticism that pornography research has received (Kohut et al., 2020; Rasmussen et al., 2018), the overall usage patterns and differences in OPU identified in questionnaire-based studies appear valid despite potential biases due to social desirability or issues related to the recall. However, we must recognize that our research could have similar limitations as self-report data concerning social desirability. Participants that are less willing to disclose their pornography use in questionnaires can also be assumed to be more likely to turn off the web tracking during their dedicated OPU sessions. Though, substantial shares of OPU in our tracking data suggest that many participants become unaware of the tracking or do not care about their OPU being tracked in many cases (at least not enough to turn off the tracking actively). Generally, it is reasonable to assume that participants become used to tracking over time.

Notably, despite the considerable similarities, there are a few specific differences between Martyniuk and Dekker's (2018) and our findings. Overall, our participants show somewhat lower levels of pornography use. We believe this general difference arises due to our focus on OPU and our operationalization of it. We also assume this focus on OPU (instead of pornography use in general) to be the main reason why, unlike in the study by Martyniuk and Dekker (2018), the youngest cohort in our study has the highest share of OP users. Other reasons for these (minor) discrepancies include differences in the sample composition and the time in which the data were collected (as it can, e.g., be assumed that OPU has further increased compared to other forms of pornography consumption over the years). In line with the results of Martyniuk and Dekker (2018) and countless other studies, our tracking data illustrate that men show more OPU than women. However, an insight that our tracking data add due to their higher granularity is that men also show much higher variation in OPU than women.

## Part Two: Correlational Analysis of Online Pornography Use Predictors Based on Linked Survey and Web Tracking Data

Several studies have looked at predictors of pornography use. The main positive predictors that have been identified include male sex, possibly a younger age, hedonistic motives, such as boredom reduction and pleasure-seeking, and low religiousness (Grubbs et al., 2019; Martyniuk & Dekker, 2018; Price et al., 2016; Štulhofer et al., 2022). Furthermore, being in a relationship might affect pornography use as, for instance, one study found that women seem to be more prone to watch pornography with a partner compared to men (Morgan, 2011). This effect of relationship status would suggest that having a partner could enhance women's pornography use. Conversely, men seem to use pornography far more often alone than women (Morgan, 2011).

Research suggests that male masturbation behavior is affected by relationship status (Baker & Bellis, 1995), which also has implications for pornography use. In a recent study, Grubbs et al. (2019) reviewed over 130 studies and, apart from the already mentioned predictors, identified individual differences in personality traits, such as sensation seeking, self-control, or narcissism, to be relevant predictors of pornography use. Sexist attitudes have also been discussed as possible predictors. However, recent studies found an inverse relationship between pornography use and sexism (Kohut et al., 2016; Speed et al., 2021). Contrary to prior expectations, these studies suggest that pornography users may be less sexist and hold more gender-egalitarian views than non-users.

The correlational analysis in the second part of our study aimed to investigate predictors of OPU and the differences therein. In addition to primary demographic attributes, such as sex and age, which we also looked at in the first descriptive part of our study, we were also particularly interested in attitudinal factors. Overall, we assessed the following predictors: primary sociodemographic data (sex, age), relationship status (Baker & Bellis, 1995; Morgan, 2011), religiosity (Short et al., 2015; Štulhofer et al., 2022), sub-facets of sexism (Kohut et al., 2016; Speed et al., 2021), and social dominance orientation (Küpper & Zick, 2011). Our first research question (RQ1) for this study is which of these factors are (the strongest) predictors of OPU as a binary variable (usage yes/no). In addition, we were also interested in assessing which of those variables is best suited for explaining variance in the frequency of OPU among users (RQ2).

### Method

#### Participants

We used the same web tracking data as in the first part but augmented it with data from an online mulitpurpose survey, originally not aiming at pornography, conducted among the web tracking sample. We invited all participants of the web tracking panel who were active in May 2019 to complete an online questionnaire. The survey was fielded from May 22 to June 3, 2019. A total of 1315 (48.1% female) participants aged 18–87 (*M*_age_ = 45.33, *SD*_age_ = 12.61) completed the questionnaire. Notably, no tracking data were available for 23 of the survey respondents.^1^

#### Measures and Analyses

The first block of predictors contains sociodemographic characteristics. Our first predictor is sex because existing research (e.g., Martyniuk & Dekker, 2018) and the first part of our study showed a significant difference in OPU between women (= coded as 1 in our data) and men (coded as 0). Second, we included age in years in the model because the first part of our study as well as other previous studies (Martyniuk & Dekker, 2018; Price et al., 2016), have shown that internet pornography use is more prevalent in younger age groups. Our web tracking data and other existing survey data (Martyniuk & Dekker, 2018) also indicate that the difference between men and women is substantially smaller in younger age groups.

The interaction effect models change linearly in sex differences depending on age. For this reason, we also included an interaction term between sex and age in the regression model after age was mean-centered. Third, we included two dummy variables for respondents that are currently single or living in a relationship without sharing a household (also called living apart together (Levin, 2004) because pornography consumption should be affected by relationship status, with singles showing higher levels of OPU (Morgan, 2011). Participants in a relationship sharing a household with their partners serve as the reference category for this variable.

In addition to primary demographic attributes, we also included further predictors in our models. The second block of predictors in our analyses contains measures for individual religiosity because religiosity is a key predictor of pornography use (e.g., Short et al., 2015; Štulhofer et al., 2022). We used a question on subjective religiosity (“How religious would you say you are?”; 1 = “not religious at all” to 7 = “very religious”), which is a parsimonious measure of the general intensity of individual religiosity (Huber & Huber, 2012).^2^ In addition, we used a question on denominational membership to measure religious identity. The sample is mainly composed of individuals without a religious affiliation (48%), German Protestants, including members of Free-Churches (24 and 3.5% respectively), and Roman Catholics (20%). Other denominations, including Muslims and Orthodox Christians, comprise only 4% of the sample. Individuals without religious affiliation are the reference category in our subsequent analyses.

The third block of predictors includes the social attitudes assumed to be associated with OPU. Because we collected the data as part of a multi-topic survey, we could not field the complete scales but instead selected a subset of items from each scale. The items are then used as indicators to create continuous latent variables by confirmatory factor analysis (see below).

As the first measure of sexism, we selected four items from a German version of the hostility dimension of the Ambivalent Sexism Inventory (ASI-Hostility) (Eckes & Six-Materna, 1999). The inventory includes a hostility dimension that best aligns with the hypothesis that pornography use can be driven by misogyny, which has, however, been somewhat refuted by recent research (Kohut et al., 2016; Speed et al., 2021). We used four items in the survey from the existing German versions of the ASI-Hostility scales (Eckes & Six-Materna, 1999).

The second measure for sexism in our study was the German language Modern Sexism Scale (MSS) (Eckes & Six-Materna, 1998). Contrary to general hostility towards women, the MSS measures subtle and hidden prejudice against women. We selected five items from the original 10-item scale that cover different social domains (e.g., labor market, media) where women experience discrimination. The choice of these items from the full scale was guided by several criteria: high item discrimination and limited distribution skewness.

To measure social dominance orientation (SDO), we used six items from a German version of an established SDO scale (Six et al., 2001). As for the MSS, the choice of items was based on limiting the skewness of distribution and maximizing item discrimination.

We used confirmatory factor analysis (CFA), widely used in psychology, to model the sexism and SDO scales as continuous latent variables. This technique uses the shared variances of the observed variables to model an unobserved latent variable. The underlying idea is that the observed indicators are causally influenced by the latent variable, representing a measurement of the latent construct. CFA reduced the measurement by using several indicators per latent construct (Hox, 2021). A valid measurement has to provide a satisfactory model fit (see below). The four scales were estimated in a single CFA to test if the survey participants distinguish the four constructs. Each scale (e.g., set of indicators) should form one latent variable. Details of the measurement are reported in Table A4 and A5 of the online Appendix).

The ASI-Hostility and MSS scales match the expected unidimensional model. Two items of the MSS are phrased negatively (see items MSS 4 and 5 in Appendix 1), which results in correlated residuals in the CFA (the correlation captures the differences in the question format). Modeling the SDO items as a single latent variable produced a significant misfit in the model. As already reported by Six et al. (2001), the items cover two different dimensions of social dominance orientation: One latent factor measures group dominance (SDO-dominance) the other a preference for group equality (SDO-equality). We estimated the CFA for all four scales in a single model. The fit indices of the measurement part of the model reported in Table 1 are satisfactory. The RMSEA value should be below 0.08, and TFI and CLI should be around 0.95 (Hu & Bentler, 1999; Xia & Yang, 2018), which indicates that the model appropriately represents the data. Roughly, the fit measures follow the logic of Chi-square testing but account for model complexity and sample size. They indicate how far data generated based on the model parameters deviate from the observed data but differ in how they account for model complexity. Therefore, we used the model with four latent variables for the subsequent analysis. The online supplementary materials report all CFA measurement parameters (e.g., factor loadings, etc.) (see Table A3 of the online Appendix).

**Table 1:** Fit measures for the measurement part of the structural equation model (n = 1286)

Fit measure	Estimate
RMSEA	0.056
CFI	0.954
TLI	0.942
SRMR	0.047

*RMSEA* root mean square error of approximation, *CFI* Comparative Fit Index, *TLI* Tucker-Lewis-Index, *SRMR* standardized root mean square residual

Our primary analysis for this study part proceeded in two steps. First, we created a binary dependent variable from the web tracking data differentiating whether study participants had visited a website disseminating sexually explicit content over the 12-month observation period. 48.5% of the survey sample visited a pornographic from the list described in the methods section for the first part of our study during the observation period (68% of the male and 28% of the female respondents). In the second step, we focused on OP users and used the average number of sessions per month as our outcome variable.

### Results

**Fig. 4:**
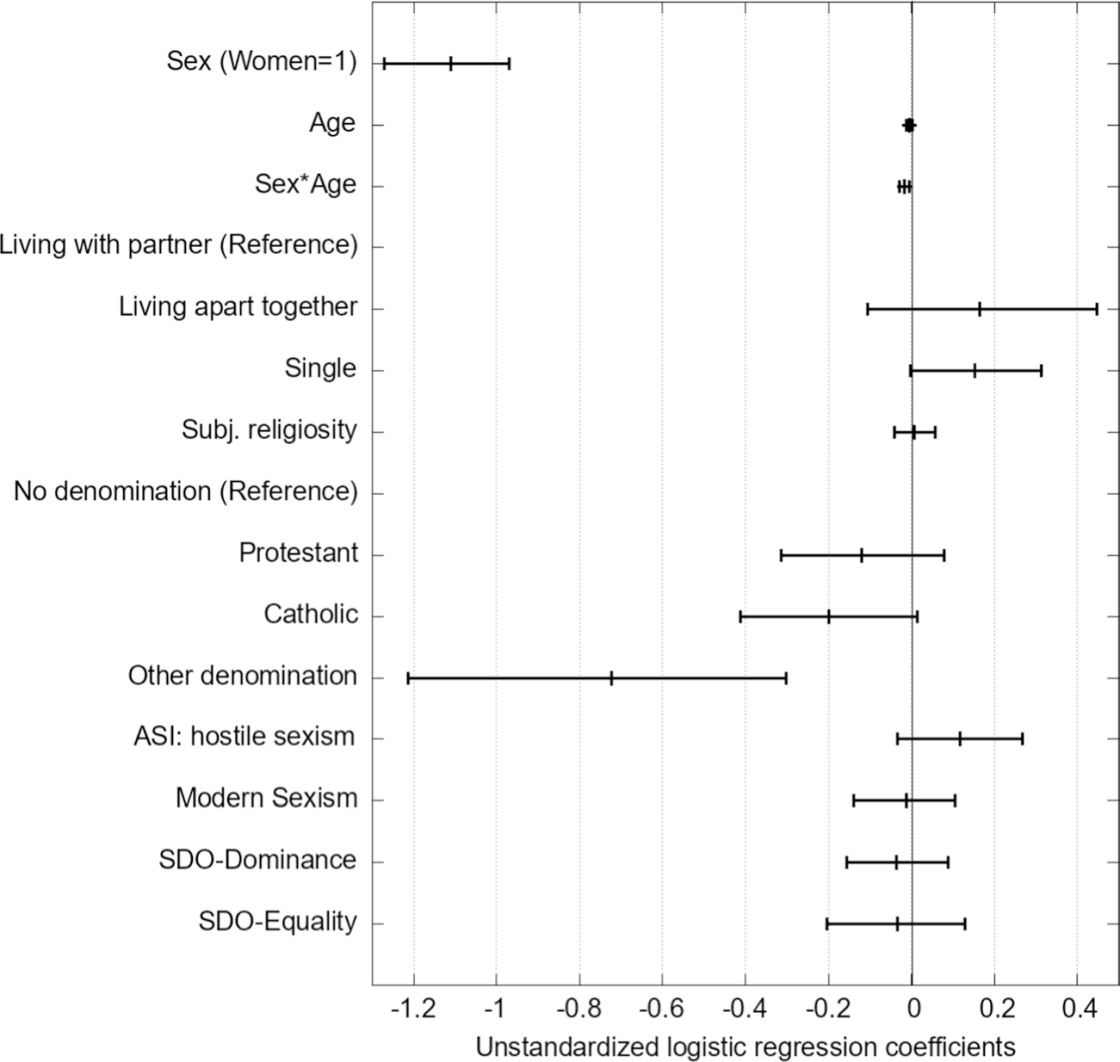
Results of the logistic regression model of the probability of opu with bootstrapped 95% confidence intervals (n = 1280). *Note.* RMSEA = 0.042, SRMR = 0.06, CFI = 0.87, TLI = 0.85. For explanations of acronyms, see the note for Table 1

**Fig. 5:**
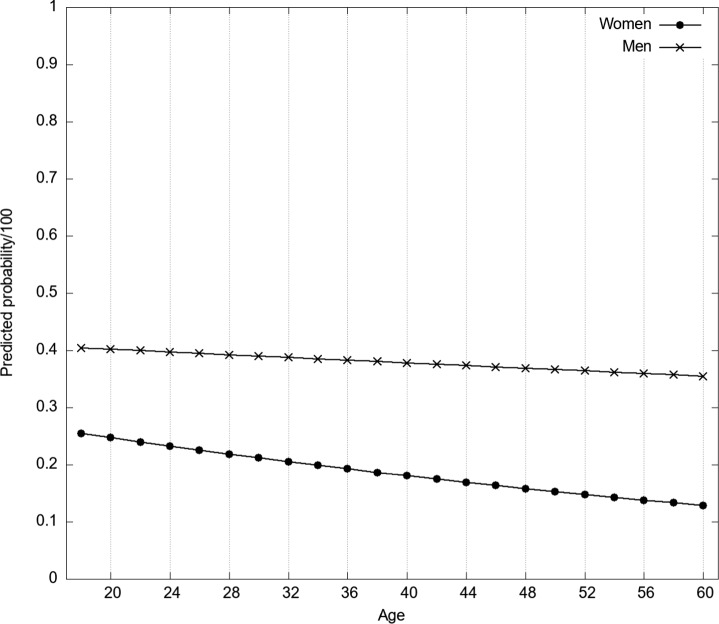
Predicted probabilities for OPU of women and men depending on age. *Note.* The probabilities were predicted for mean values of continuous covariates (ASI, MSI, etc.), Catholics, and singles

**Fig. 6:**
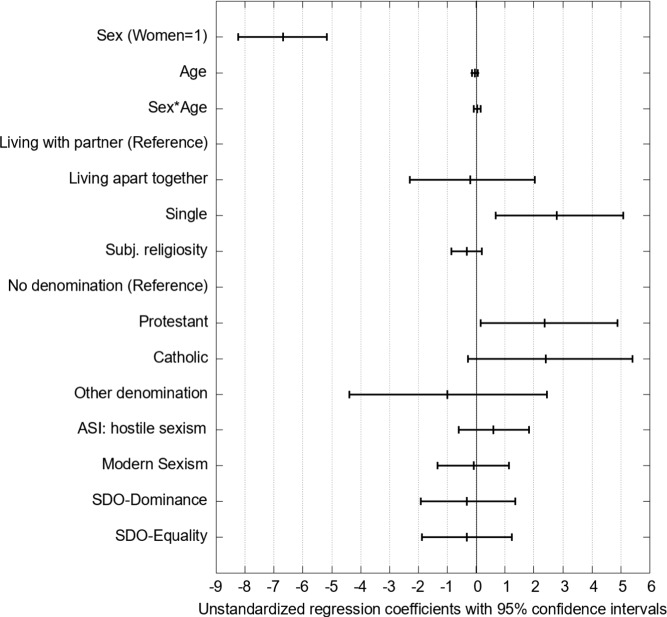
Results of the regression model of the frequency of online pornography use with bootstrapped 95% Confidence Intervals (n = 622). *Note.* RMSEA = 0.04, SRMR = 0.052, CFI = 0.94, TLI = 0.93. For explanations of acronyms, see note for Table 1

We ran a logistic regression with WLSMV estimation to answer our first research question (Li, 2016).^3^ Figure 4 reports the unstandardized regression coefficients with 95%-confidence intervals. We used 5000 bootstrap runs to estimate the confidence intervals for Figs. 4 and 6. Our results show that women are significantly less likely to visit pornographic websites (*b* = − 1.092, *p* < .001 when controlling for sociodemographic, religiosity, and gender-related attitudes. We do not find an effect of age for men (*b* = − 0.005, *p* = .225). We find a significant interaction between age and sex (*b* = − 0.015, *p* = .010). The effect of age is significant for women (*b* = − 0.021, *p* < .001). Whereas there are only small age differences in OPU for men, younger women used OP more often than older women. Because the logit coefficients are challenging to interpret, Fig. 5 shows the predicted probabilities of OPU for men and women depending on age. The probability is systematically higher for men and decreases only very slightly with age. Women have a lower predicted probability of OPU that decreases from about 25% in the youngest age to about 12% at age 60.

Hence, overall, the survey study confirms the descriptive result from our exploratory analysis: the difference in OPU between women and men increases with age. The regression also suggests that singles are slightly more likely to visit pornographic websites than persons living in a household with their partners. However, this effect is not significant (*b* = 0.156, *p* = .082).

The measure for individual religiosity is not associated with the probability of OPU (*b* = 0.006, *p* = .822). However, all members of religious denominations have a lower probability of OPU compared to the unaffiliated. Still, this difference is neither significant for Catholics (*b* = − 0.184, *p* = .090) nor German Protestants (*b* = − 0.117, *p* = .240). A strong and significant negative effect is visible for members of the minority religions in the sample (e.g., Islam, Orthodox Christians; *b* = − 0.715, *p* = .002).

The two sexism scales and the two dimensions of social dominance orientation were unrelated to the probability of OPU. Only for the ASI Hostility scale, a slightly positive effect was visible which, however, was not significant (*b* = 0.111, *p* = .135). We also estimated the models separately for women and men, but the results are very similar for both sexes (as reported in Models 1c and 1d of Table A6 of the online Appendix). We also tested for potential non-linear associations between age and OPU as well as religion and OPU by including dummies for age categories and different levels of the subjective religiosity scale (reported in Model 1b of Table A6 of the online Appendix 4) but did not find evidence for non-linear associations. Overall, the model provides a good fit for the data. 27% of the variance of the dependent variable is explained by the predictors, with sex explaining by far the largest proportion of the variance.^4^

Our second analysis focuses on the frequency of OPU as the outcome variable. We only included respondents who visited at least one pornographic website over the observation period (*n* = 622). The dependent variable is the average number of OPU sessions per month (*M* = 6.95, *Mdn*. = 2.33, *Min* = 0.08, *Max* = 105.42), and we use the same predictors as in the first analysis. The estimates reported in Fig. 6 are unstandardized linear regression coefficients with 95%-confidence intervals (the online Appendix reports the coefficients and standard errors).

The strong effect of sex also exists for the frequency of OPU. The model estimates that men have about seven pornographic web sessions per month compared to women (*b* = − 6.636, *p* < .001).^5^ Again, there is no effect of age for men. For this model, the interaction of age and sex is not significant. Within the group of women using OP, age is unrelated to differences in OPU frequency. The reason for this pattern is that women visiting pornographic websites are younger on average. Singles have more OP sessions than individuals living in relationships (*b* = 2.620, *p* = .007).

From the religion variables, only Protestant church members had more OP sessions (*b* = 2.331,* p* = .045), but the effect is only borderline significant.

Similar to the first analysis, the scales measuring sexism and social dominance orientation are unrelated to the frequency of OPU. Overall, the predictors we included in the second model explain about 5% of the variance of OPU frequency. In sum, the predictive power of the first model (OPU binary) is much higher than that of the second model (OPU frequency). Models using dummies for age categories and religiosity and separated for men and women are available in Table A7 of the online Appendix.

### Discussion

Augmenting the web tracking with survey data enabled us to study how individual-level predictors are related to the probability of using OP and the frequency of OPU. Similar to the first part of our study, our findings replicate major results from prior survey-based research. First and foremost, our data show that different facets of sexism and social dominance orientation do not predict pornography use. Furthermore, being of Christian faith seems to be, given the effect sizes, not very strongly related to pornography use. However, we would not attribute this to a bias in prior research (e.g., Short et al., 2015; Štulhofer et al., 2022) but rather to more liberal moral views of Christians in Germany compared to the US, where widespread evangelical congregations defend more restrictive sexual morality (Edger, 2012). Another explanation for our finding could be that Christians have a higher propensity to mute web tracking when they visit pornographic websites due to the religious prohibition of sexually explicit media. In line with previous research and the first part of our study, the results of the regression models confirm that the male sex is the primary predictor of pornography use.

Contrary to our assumptions, relationship status does not significantly predict OPU but only the frequency of OPU. Overall, the model results differ only slightly between women and men. OPU for women depends on age which is different for men.

Even when controlling for basic sociodemographic attitudes, religious variables, and social attitudes towards women, sex has—by far—the most potent effect on both probability of OPU and the frequency of OPU. The logistic regression model also confirmed another thing we found in our descriptive analyses: the sex difference increases with age. Notably, as our data are cross-sectional, we do not know whether this result stems from an age effect because older women become less likely to use OP or whether sex differences are smaller in younger generations. The latter seems more likely, given the trend toward more permissive sexual morality and female empowerment in Western societies (Alexander et al., 2016).

From a methodological point of view, the linked data requires different analysis methods because the web tracking is count data that is typically skewed. For that reason, we decided to split the analysis into two parts: In the first part, we looked at the probability of OPU as a binary outcome (yes/no), and in the second part, we used the frequency of OPU as our outcome of interest. On the one hand, we assumed that the process explaining the probability of OPU differs from the prediction of OPU frequency. This assumption, however, was not confirmed by our analysis. On the other hand, the two-part analysis avoids influential cases, heavily biasing the analysis results.

## General Discussion

The first aim of our two-part study was to replicate the findings on PU usage patterns and differences identified for Germany by Martyniuk and Dekker (2018) while using a more finely-grained data source. Using web tracking data, we replicated the differences in age and sex identified in previous survey research. Despite recent criticisms of the field of pornography research (Kohut et al., 2020; Rasmussen et al., 2018), our second study confirms and somewhat extends many of the key findings from previous (survey-based) research. Sex and age remain the strongest predictors of OPU, indicating a strong biological drive, at least in males, possibly linked to masturbation behavior and sex drive (Baker & Bellis, 1995; Lippa, 2009; Morgan, 2011). Religiousness, which some researchers have characterized as a potential cultural taming force of nature (Pinker, 2003), somewhat inhibits OPU in our second study regarding small religious congregations but not the mainstream Christian churches in Germany. Specifically, in our sample, OPU was lower for members of religious minority groups than those without a religious denomination. However, in the case of Protestants, we also found that the members of this group who use OP do so with a higher frequency than others. Similar to several previous studies (Kohut et al., 2016; Speed et al., 2021), our analysis did not find associations between sexism and other attitudinal variables, such as social dominance orientation, with OPU. In summary, our studies demonstrate that key findings from research exclusively based on self-reports can be replicated with tracking data and tracking data combined with survey data. Nevertheless, we still see advantages in using tracking data and combining it with surveys as they are arguably more objective, less prone to social desirability bias, and allow for more finely-grained analyses, in particular, longitudinal ones with higher temporal solutions (Kohut et al., 2020; Rasmussen et al., 2018).

### Study Limitations

While using web tracking data in combination with survey data provides several advantages compared to only using self-reported data, our study had many limitations to consider when interpreting its results. To begin with: the data originates from a non-probability sample in which users agree to have their internet use tracked. This sample does not represent the German population (or even the population of German internet users). For example, participants in the tracking sample were, on average, younger and better educated than the general population in Germany. Also, we only had data from Germany, so the question remains open to what degree our findings are generalizable to other countries.

A limitation of the tracking data is that participants could pause the tracking, and some of the measured attitudes may be related to using this possibility when consuming OP. Hence, we cannot completely rule out that processes of social desirability may have also influenced our web tracking data. However, compared to self-reported survey measures, pausing web tracking requires a conscious effort from study participants. Therefore, we are still convinced that the non-intrusive measurement of OPU is, overall, more reliable than self-reported behavior. Notably, using tracking data does not solve the question of scale. As we have discussed and shown, just like for self-report data, OPU can be operationalized in different ways with tracking data. We used a specific definition of a usage session and used counts of these instead of duration as our key outcome variable. Also, we focused on video, photo, and live cam sites in our analyses. Another limitation in this context is that we only have data on the domain level, which makes it impossible to distinguish in more detail between different types of pornographic content. Notably, what is typical of interest is not the absolute value of (some measure of) OPU but the relative differences and changes in OPU. The latter is stable across different operationalizations of OPU (as also illustrated by the correspondence between our findings and previous ones based on self-report data). The survey data we collected also has limitations that need to be considered. The scales used for our analysis do not contain all items but are a selection from the original instruments. The CFA, however, has confirmed the high quality of the measurement.

Moreover, we used a parsimonious measurement of individual-level religiosity. While it might be the case that different ways of being religious [e.g., literal beliefs (Upenieks, 2022), intrinsic religiosity (Stavrova & Siegers, 2014)] are differently related to OPU. Recent studies have also shown that the effects of religiosity might be mediated by psychological traits (Borgogna et al., 2020).

## Conclusion

Following the call by Grubbs and Kraus (2021), our aim with this paper was to assess the reliability of prior findings on OPU that have been criticized for solely relying on self-reported data (Kohut et al., 2020; Rasmussen et al., 2018). While previous research exists that has used web-tracking data to study OPU (e.g., Lewczuk et al., 2022; Morichetta et al., 2021), we combined objective web-tracking with survey data and can test the influence of attitudes and traits on OPU, which would not be possible without a mixed-methods approach. Furthermore, we used data on an individual level different from the one an internet provider can offer. Despite the justified criticism regarding pornography research, our analyses indicate that the key findings from previous research based on self-reported data seem reliable. Sex and age were significant predictors of pornography use. Religion can have a diminishing effect on smaller congregations, though some religious individuals who use pornography are prone to watch more pornography. However, this effect was small. Relationship status generally did not predict the probability of OPU but the frequency with singles having more OP sessions per month. Also, similar to several previous studies (Kohut et al., 2016; Speed et al., 2021), neither sexism nor social dominance orientation predicted OPU in our sample.

Considering the limitations of our study and the questions that remain to be answered, a worthwhile avenue for future research could be using more detailed web tracking data (e.g., using complete URLs instead of domains). Studies using respective data could combine content analysis with tracking information and survey data. Such a research design would allow for explaining specific OPU patterns or preferences for specific pornographic content. Furthermore, web tracking data (in combination with survey data) could also be used to study the formation or breaking of OPU habits or potentially problematic OPU, especially a high intensity of usage, in more detail. Another valuable next step for OPU research with tracking data would be comparative research with data from different countries. Combinations of web tracking and survey data could be used to test the validity and reliability of different self-report measures to contribute to the improvement of methods used in pornography research, as has been called for by several authors (Kohut et al., 2020; Marshall & Miller, 2019; Rasmussen et al., 2018).

Finally, web-tracking data are better suited than survey data to study the formation of OPU habits and user profiles regarding content preferences. Future research should investigate if temporal patterns at the individual level can be linked to study participants' social and psychological characteristics, including other forms of online activities. This research could also help develop better preventive measures against excessive OPU. Studying content preferences would require more data on OP exposure that is currently not available because—different from online news—systematic content databases are lacking.

### Supplementary Information

Below is the link to the electronic supplementary material.

Supplementary file1 (PDF 242 kb)

Supplementary file2 (CSV 277 kb)

Supplementary file3 (PDF 53 kb)

Supplementary file4 (XLSX 827 kb)

## Data Availability

The data is available via an OSF repository: 10.17605/OSF.IO/6RC35.

## Footnotes

1 There are multiple possible reasons for the lack of tracking data for these respondents. These participants may have been recruited into the panel only briefly before the field phase of our survey started, so no tracking data had been generated by them prior to the survey. Another possible explanation is that these participants turned off the tracking or did not use the tracked devices in the months for which we purchased the tracking data. Unfortunately, with the data we have, we cannot identify the individual reason for each participant.

2 All item wordings from the survey and descriptive statistics are reported in the online Appendix (see Tables A1, A2, and A3).

3 The Weighted Least Squares with Means and Variances adjusted (WLSMV) estimator creates and analyzes a matrix of polychoric, tetrachoric, and/or polyserial correlations and an associated weight matrix (Asparouhov & Muthén 2010); Muthén & Muthén 2014.

4 In logistic regressions with WLSMV estimation the R2 is the explained variance proportion of a continuous underlying latent response variable. Details on the estimation are given by Muthén and Muthén (1998–2014).

5 The intercept for average monthly visits is 202. This means that—everything else being equal—men have about 200 monthly visits and women about 30.

## Code Availability

Analyses scripts (R, SPSS, and MPlus code/syntax) are available via an OSF repository: 10.17605/OSF.IO/6RC35.
