# Supplementary material for: A Re-evaluation of Online Pornography Use in Germany: A Combination of Web Tracking and Survey Data Analysis
Source: Arch Sex Behav. 2023 Aug 29;52(8):3491–503. doi: 10.1007/s10508-023-02666-8 (PMC10703962; doi:10.1007/s10508-023-02666-8)
Supplement: Supplementary file 1 — Supplementary file1 (PDF 242 kb) [file 10508_2023_2666_MOESM1_ESM.pdf]

Tables A1: *Overview of question wordings inclusive translation from the web survey*

Variable name	German question wording	English translation
MSS 1	Diskriminierung von Frauen ist in Deutschland immer noch ein Problem.	Discrimination against women is still a problem in Germany.
MSS 2	In den Medien gibt es häufig frauenfeindliche Darstellungen.	There are often misogynistic representations in the media.
MSS 3	Frauen und Männer haben in der heutigen Gesellschaft die gleichen Chancen, etwas zu erreichen.	Women and men have equal opportunities to succeed in today's society.
MSS 4	Heutzutage werden Frauen im Berufsleben fair behandelt.	Today, women are treated equally in professional life.
MSS 5	Frauen finden häufig keine gut bezahlte Arbeit, weil sie diskriminiert werden.	Women often cannot find good paying jobs because they are discriminated against.
ASI Hostility 1	Wenn Frauen in einem fairen Wettbewerb gegenüber Männern den Kürzeren ziehen, behaupten sie gerne, sie seien diskriminiert worden.	When women lose out to men in a fair competition, they like to claim they have been discriminated against.
ASI Hostility 2	Viele Frauen interpretieren harmlose Äußerungen oder Handlungen als frauenfeindlich.	Many women interpret harmless statements or behaviours as misogynistic.
ASI Hostility 3	Frauen übertreiben im Allgemeinen die Probleme, die sie am Arbeitsplatz mit Männern haben.	Women generally exaggerate the problems they have with men in the workplace.
ASI Hostility 4	Viele Frauen haben Spaß daran, mit Männern zu spielen, indem sie sich zuerst verführerisch geben, dann aber die Annäherungsversuche der Männer zurückweisen.	Many women enjoy playing with men by first pretending to be seductive but then rejecting men's advances.
SDO dominance 1	Wenn bestimmte Gruppen unter sich bleiben würden, hätten wir weniger Probleme.	If certain groups stayed among themselves, we would have less problems.
SDO dominance 2	Es ist wahrscheinlich ganz gut, dass bestimmte Gruppen in der Gesellschaft oben stehen und andere unten.	It's actually probably quite good that certain groups are at the top of society and others at the bottom.
SDO dominance 3	Unterlegene Gruppen sollten unter sich bleiben.	Inferior groups should stay among themselves.
SDO Equality 1	Wir sollten unser Möglichstes tun, um die Bedingungen für die unterschiedlichen Gruppen anzugleichen.	We should do our best to equalise the conditions for the different groups.
SDO Equality 2	Wir hätten weniger Probleme, wenn wir alle Gruppen gleich behandeln würden.	We would have fewer problems if we treated all groups equally.
SDO Equality 3	Soziale Gleichheit sollte zunehmen.	Social equality should increase.
Sex	Bitte geben Sie Ihr Geschlecht an.	Please enter your sex.
	0=Mann	0=Male
	1=Frau	1=Female
Age	Würden Sie uns sagen, in welchem Jahr Sie geboren sind?	Would you tell us in which year you were born?
Self-rated religiosity	Unabhängig davon, ob Sie einer Kirche oder Gemeinde angehören, würden Sie von sich sagen, dass Sie eher	Regardless of whether you belong to a church or congregation, would you say of yourself that you are rather
	religiös oder eher nicht religiös sind?	religious or rather not religious?
	1=gar nicht religiös	1=not religious at all
	7=sehr religiös	7=very religious

**Variable name**	**German question wording**	**English translation**
Denomination	Welcher Konfession oder Glaubensgemeinschaft gehören Sie an?	Which denomination or religious community do you belong to?
	1 der evangelischen Kirche (ohne Freikirchen)	1 the Protestant church (without free churches)
	2 einer evangelischen Freikirche	2 a Protestant free church
	3 der römisch-katholischen Kirche	3 the Roman Catholic Church
	4 einer anderen christlichen Religionsgemeinschaft (z.B. einer	4 another Christian denomination (e.g. an Orthodox church)
	Orthodoxen Kirche)	Orthodox church)
	5 einer muslimischen Gemeinde	5 a Muslim community
	6 einer anderen nicht-christlichen Religionsgemeinschaft	6 another non-Christian religious community
	7 keiner Religionsgemeinschaft	7 No religious community
Marital Status	Welchen Familienstand haben Sie?	What is your marital status?
	1 Verheiratet und leben mit Ihrem Ehepartner zusammen	1 Married and living with your spouse
	2 Verheiratet und leben getrennt	2 Married and living separately
	3 Verwitwet	3 Widowed
	4 Geschieden	4 Divorced
	5 Ledig	5 Single
	6 Eingetragene Lebenspartnerschaft, zusammenlebend	6 Registered civil partnership, living together
	7 Eingetragene Lebenspartnerschaft, getrennt lebend	7 Registered civil partnership, living separately
	8 Eingetragener Lebenspartner verstorben	8 Registered civil partner deceased
	9 Eingetragene Lebenspartnerschaft aufgehoben	9 Registered civil partnership dissolved
Relationship status	Haben Sie einen festen Lebenspartner?	Do you have a life partner?
	1 Ja	1 Yes
	2 Nein	2 No
Shared Household	Leben Sie mit Ihrer Partnerin oder Ihrem Partner in einem gemeinsamen Haushalt?	Do you live with your partner in a shared household?
	1 Ja	1 Yes
	2 Nein	2 No

Table A2: *Overview of descriptive statistics of ordinal and metric variables*

Variable name	Mean	Variance	Min	Max
MSS 1	2.415	1.159	1	5
MSS 2	2.901	1.143	1	5
MSS 3	3.109	1.222	1	5
MSS 4	2.931	1.006	1	5
MSS 5	2.728	1.083	1	5
ASI Hostility 1	3.011	1.005	1	5
ASI Hostility 2	3.099	1.051	1	5
ASI Hostility 3	2.773	1.045	1	5
ASI Hostility 4	2.855	1.195	1	5
SDO dominance 1	2.563	1.265	1	5
SDO dominance 2	2.403	1.182	1	5
SDO dominance 3	2.217	1.114	1	5
SDO Equality 1	3.640	1.026	1	5
SDO Equality 2	3.516	1.157	1	5
SDO Equality 3	3.910	1.193	1	5
Age	45.335	12.6	18	87
Self-rated religiosity	2.735	3.169	1	7

Table A3: *Distribution of categorical and nominal variables*

**Sex**	
Men	51.8
Women	48.2
**Denomination**	
Protestants (incl. Free Churches)	27.1
Roman Catholic	20.8
Other denomination	3.6
No denomination	48.3
**Relationship status**	
Relationship with shared household	57.8
Living apart together	8.3
Single	33.9

Table A4: *Measurement parameters of the Confirmatory Factor Analysis*

	λ	s.e.
**Modern Sexism Inventory**		
SEXISM2	0.827	0.016
SEXISM3	0.611	0.021
SEXISM4B	0.372	0.027
SEXISM5B	0.480	0.025
SEXISM6	0.781	0.017
**Hostile Sexism (from Ambivalent Sexism Inventory)**		
ASI1	0.744	0.017
ASI2	0.782	0.015
ASI3	0.792	0.016
ASI4	0.652	0.019
**Social Dominance Orientation – Group Dominance**		
SDO4	0.692	0.019
SDO2	0.721	0.018
SDO6	0.838	0.015
**Social Dominance Orientation – Group Equality**		
SDO1	0.864	0.015
SDO3	0.708	0.018
SDO5	0.722	0.018

Table A5: *Correlations between latent variables measuring gender related attitudes*

	Modern Sexism Inventory	ASI Hostile Sexism	SDO-Group Dominance	SDO-Group Equality
**Modern Sexism Inventory**	1	0.387	0.192	-0.205
**ASI Hostile Sexism**		1	0.369	-0.106
**SDO-Group Dominance**			1	-0.546
**SDO-Group Equality**				1

Table A6: *Overview of logistic regression models for online pornography use*

	Modell 1a (Reported model)		Model 1b (dummies for age and religion)		Model 1c (Model 1a for men only)		Model 1d (Model 1a for women only)	
	b	s.e.	b	s.e.	b	s.e.	b	s.e.
ASI: hostile sex.	0.111	0.074	0.106	0.071	0.101	0.102	0.119	0.099
Modern Sexism	-0.013	0.061	-0.014	0.059	0.005	0.073	-0.055	0.093
SDO-Dominance	-0.030	0.063	-0.032	0.058	-0.024	0.083	-0.048	0.089
SDO-Equality	-0.021	0.082	-0.026	0.077	-0.037	0.099	-0.007	0.118
Sex	-1.092***	0.076	-1.175***	0.310	-	-		
Age	-0.005	0.004	-	-	-0.005	0.004	-0.021***	0.005
Sex*Age	-0.015**	0.006	-	-	-	-		
Age <25	-	-	Reference	-	-	-	-	-
Age 26-35	-	-	-0.251	0.281	-	-	-	-
Age 36-45	-	-	-0.334	0.282	-	-	-	-
Age 46-55	-	-	-0.163	0.277	-	-	-	-
Age >55	-	-	-0.448	0.275	-	-	-	-
Age <25*Sex	-	-	Reference		-	-	-	-
Age 26-35*Sex	-	-	0.420	0.352	-	-	-	-
Age 36-45*Sex	-	-	0.153	0.351	-	-	-	-
Age 46-55*Sex	-	-	0.086	0.337	-	-	-	-
Age >55*Sex	-	-	-0.335	0.349	-	-	-	-
Living with partner	Reference							
Living apart together	0.142	0.138	0.156	0.152	0.408	0.224	-0.077	0.196
Single	0.156	0.082	0.184*	0.084	0.203	0.114	0.085	0.121
Subj. religiosity	0.006	0.025	-	-	0.028	0.034	-0.021	0.035
Subj. rel1			Reference	-	-	-	-	-
Subj. rel2			-0.197	0.118	-	-	-	-
Subj. rel3			-0.008	0.141	-	-	-	-
Subj. rel4			-0.080	0.126	-	-	-	-
Subj. rel5			0.012	0.134	-	-	-	-
Subj. rel6			-0.025	0.164	-	-	-	-
No affiliation	Reference	-	Reference	-	-	-	-	-
Protestant	-0.117	0.099	-0.079	0.102	-0.285	0.137	0.064	0.140
Catholic	-0.184	0.108	-0.148	0.109	-0.372*	0.149	0.010	0.152
Other denom.	-0.715**	0.234	-0.711***	0.223	-0.808**	0.286	-0.642	0.350
N	1280		1280		659		618	

Table A7: *Overview of OLS regression models for frequency of online pornography use (average sessions per month dependent)*

	Modell 2a (Reported model)		Model 2b (dummies for age and religion)		Model 2c (Model 2a for men only)		Model 2d (Model 2a for women only)	
	b	s.e.	b	s.e.	b	s.e.	b	s.e.
ASI: hostile sex.	0.599	0.624	0.602	0.754	0.796	1.073	-0.149	0.461
Modern Sexism	-0.095	0.613	-0.055	0.630	-0.057	0.787	0.068	0.542
SDO-Dominance	-0.302	0.779	-0.378	0.784	-0.045	1.313	-0.718	0.459
SDO-Equality	-0.16	0.838	-0.351	0.887	-0.067	1.277	-0.998	0.518
Sex	-6.636	0.776	-8.509**	3.320	-	-	-	-
Age	-0.043	0.053	-	-	-0.039	0.051	-0.045	0.027
Sex*Age	0.039	0.063	-	-	-	-	-	-
Age <25	-	-	Reference	-	-	-	-	-
Age 26-35	-	-	-1.087	2.508	-	-	-	-
Age 36-45	-	-	-1.615	2.459	-	-	-	-
Age 46-55	-	-	-1.694	2.374	-	-	-	-
Age >55	-	-	-2.378	2.411	-	-	-	-
Age <25*Sex	-	-	Reference		-	-	-	-
Age 26-35*Sex	-	-	1.785	3.914	-	-	-	-
Age 36-45*Sex	-	-	2.113	3.992	-	-	-	-
Age 46-55*Sex	-	-	1.824	3.777	-	-	-	-
Age >55*Sex	-	-	2.054	4.297	-	-	-	-
Living with partner	Reference		Reference		Reference		Reference	
Living apart together	-0.290	1.125	-0.215	1.668	-0.098	2.289	-0.844	1.066
Single	2.620*	1.096	2.717	0.974	3.578**	1.301	-0.133	0.653
Subj. religiosity	-0.315	0.268	-	-	-0.471	0.390	0.122	0.192
Subj. rel1	-	-	Reference	-	-	-	-	-
Subj. rel2	-	-	-1.044	1.377	-	-	-	-
Subj. rel3	-	-	-2.241	1.707	-	-	-	-
Subj. rel4	-	-	-0.294	1.496	-	-	-	-
Subj. rel5	-	-	-0.391	1.516	-	-	-	-
Subj. rel6	-	-	-4.032*	1.979	-	-	-	-
No affiliation	Reference	-	Reference	-	Reference	-	Reference	-
Protestant	2.331*	1.178	2.613	1.178	3.387*	1.598	-0.485	0.742
Catholic	2.419	1.449	2.867	1.315	3.355	1.740	-0.182	0.844
Other denom.	-1.034	1.720	-0.064	3.097	-0.467	4.029	-1.406	2.317
N	622		622		450		171	
