# Supplementary material for: A Re-evaluation of Online Pornography Use in Germany: A Combination of Web Tracking and Survey Data Analysis
Source: Arch Sex Behav. 2023 Aug 29;52(8):3491–503. doi: 10.1007/s10508-023-02666-8 (PMC10703962; doi:10.1007/s10508-023-02666-8)
Supplement: Supplementary file 3 — Supplementary file3 (PDF 53 kb) [file 10508_2023_2666_MOESM3_ESM.pdf]

Domain	Number of total visits	Category
xhamster.com	383056	Video portal
planetromeo.com	337334	Casual dating
chaturbate.com	288119	Webcam portal
pornhub.com	263529	Video portal
poppen.de	150928	Casual dating
joyclub.de	140579	Casual dating
coedcherry.com	85462	Video portal
xvideos.com	74112	Video portal
youporn.com	65415	Video portal
xnxx.com	52506	Video portal
eroprofile.com	50527	Casual dating
144chan.pk	47139	Photo portal
eronity.com	35596	Casual dating
pr0gramm.com	29792	Wrongly assigned
redtube.com	29635	Video portal
hqbabes.com	27694	Photo portal
yourporn.sexy	22555	Wrongly assigned
nurxxx.mobi	19247	Video portal
nutaku.net	18402	Eroticism
txxx.com	14642	Video portal
pornpics.com	13163	Photo portal
livejasmin.com	12885	Webcam portal
amateurcommunity.com	12823	Casual dating
perfektdamen.co	12817	Video portal
eporner.com	12704	Video portal
treff6.de	12484	Casual dating
eis.de	12135	Wrongly assigned
ixxx.com	11712	Video portal
beeg.com	11669	Video portal
xhamsterlive.com	11155	Webcam portal
justporno.sex	10919	Webcam portal
my-homo.net	10528	Casual dating
storiesonline.net	10357	Eroticism
faptitans.com	10220	Eroticism
porn300.com	9838	Video portal
gotporn.com	9666	Video portal
pbwstatic.com	9454	Wrongly assigned
big7.com	9422	Video portal
brdteengal.com	9127	Photo portal
myfreecams.com	9086	Webcam portal
visit-x.net	8964	Webcam portal
cam4.com	8946	Webcam portal
tubegalore.com	8910	Video portal
tnaflix.com	8196	Video portal
planetsuzy.org	8123	Eroticism
gayforit.eu	7925	Video portal
ebenporno.com	7704	Video portal
tube8.com	7668	Video portal
fetisch.de	7613	Casual dating
hclips.com	7562	Video portal
gayboystube.com	7052	Video portal
pornohirsch.com	6911	Video portal
nudevista.com	6827	Video portal
xtube.com	6778	Video portal
boyfriendtv.com	6748	Video portal
pornmd.com	6727	Porn search engine
thesettlersonline.com	6560	Wrongly assigned
sunporno.com	6530	Video portal
hqporner.com	6447	Video portal
ashemaletube.com	6444	Video portal
vporn.com	6357	Video portal
fuq.com	6264	Video portal
pornsos.com	5883	Video portal
gaymaletube.com	5812	Video portal
rotlicht.de	5711	Prostitution

Category[1]	Explanation
Video portal	„Porn Tube“
Webcam portal	Webcam-Tube
Casual dating	Website aims at casual dating or dating in general (grey area with prostitution)
Distributor	Webpage who just publishes the links of different content providers
Producers website	Original porn-content producers
Photoportal	Website who publishes pornographic pictures predominantly
Eroticism	Either Storyblog about erotic stories, website to chat about eroticism and sexuality or game-, video and storyblog without a clear a
Porn search engine	Search engine that specifically aims at pornography
Prostitution	Website to specifically advocage prostitution etc. (legal in Germany)
Erotic cartoons or hentai portal	Portal specifically about pornographic comics and hentai
Torrent	Torrentsites for porn distribution
Wrongly assigned	Wrongly assigned pages like onlinestores, men's magazines etc.

Note. [1]The majority of the coded websites serve different purposes. Categories were assigned regarding the supposed main aime or usage purpose of the respective website.

bongacams.xxx	5530	Webcam portal
heuteporno.com	5503	Video portal
machomoe.com	5474	Video portal
pornhubdeutsch.net	5432	Wrongly assigned
heavy-r.com	5429	Video portal
maturetube.com	5426	Video portal
analdin.com	5378	Video portal
4fuckr.com	5170	Webcam portal
between-legs.com	5092	Photo portal
sexy-beauties.com	5086	Photo portal
freepornq.com	4884	Video portal
upornia.com	4830	Video portal
liebeakt.com	4685	Video portal
manhub.com	4627	Video portal
youjizz.com	4583	Video portal
nudevista.at	4510	Video portal
luscious.net	4313	Erotic cartoons or hentai portal
clubseventeen.com	4047	Producers website
nakedgirls.mobi	4030	Video portal
imagefap.com	4027	Photo portal
drtuber.com	3994	Video portal
hotmovs.com	3988	Video portal
sexuria.com	3972	Video portal
pornhd.com	3938	Video portal
wankerlab.com	3839	Video portal
iwank.tv	3789	Video portal
4porn.com	3762	Video portal
pornkino.to	3750	Video portal
pussy.bz	3735	Wrongly assigned
pictoa.com	3715	Photo portal
empflix.com	3705	Video portal
porzo.com	3700	Video portal
endloseporno.com	3649	Video portal
vrporn.com	3638	Video portal
xsexcomics.com	3413	Erotic cartoons or hentai portal
tubepleasure.com	3390	Video portal
daftsex.com	3390	Video portal
nacktsonnen.com	3375	Photo portal
apornstories.com	3358	Eroticism
owl-intim.de	3323	Prostitution
sxyprn.com	3309	Video portal
jizzbunker.com	3233	Video portal
spankwire.com	3216	Video portal
porndroids.com	3151	Video portal
rule34.xxx	3151	Porn search engine
bongacams.com	3122	Webcam portal
bobolike.com	3121	Video portal
hdzog.com	3120	Video portal
nur.xxx	3115	Video portal
hd-pornos.net	3109	Video portal
porn.com	3068	Video portal
youporndeutsch.xyz	3053	Video portal
cuntwars.com	2952	Eroticism
perversefrage.com	2934	Eroticism
nuvid.com	2903	Video portal
mature.nl	2899	Video portal
cooch.tv	2877	Video portal
youporngay.com	2846	Video portal
playboy.de	2838	Eroticism
adult-empire.com	2834	Producers website
erocurves.com	2828	Photo portal
stripchat.com	2780	Webcam portal
dianapost.com	2776	Photo portal
foxporns.com	2753	Video portal
3dsex.pics	2717	Photo portal
tube188.com	2716	Video portal
a1tb.com	2651	Video portal

alohatube.com	2635	Video portal
whatsexy.de	2630	Casual dating
reif6.com	2624	Casual dating
pornxs.com	2613	Video portal
amandalist.com	2603	Photo portal
xl-gaytube.com	2577	Video portal
bravotube.net	2562	Video portal
sirporno.xxx	2557	Video portal
xxx3dcomix.com	2540	Erotic cartoons or hentai portal
watchxxxfreeinhd.com	2518	Video portal
ice-gay.com	2515	Video portal
sexu.com	2494	Video portal
6mature9.com	2461	Photo portal
amateurcommunity.de	2456	Casual dating
hostessen-meile.com	2443	Prostitution
yespornplease.com	2439	Video portal
xcafe.com	2432	Video portal
scharfecams.com	2426	Webcam portal
thisvid.com	2412	Video portal
porndoe.com	2352	Video portal
porngals4.com	2291	Photo portal
amarotic.com	2283	Producers website
extremetube.com	2243	Video portal
beautyprettyteen.com	2221	Photo portal
german-porno-deutsch.info	2220	Video portal
anyporn.com	2218	Video portal
sexy3dcomix.com	2199	Erotic cartoons or hentai portal
keezmovies.com	2191	Video portal
gayporno.fm	2189	Video portal
pornojux.com	2143	Video portal
serviporno.com	2126	Video portal
pornmarathon.com	2109	Video portal
cumlouder.com	2068	Video portal
professor-porno.com	2012	Producers website
boy18tube.com	2004	Video portal
pornoente.tv	1984	Video portal
mymusclevideo.com	1982	Video portal
freeporncategories.com	1971	Video portal
tubev.sex	1971	Video portal
lobstertube.com	1970	Video portal
pornorc.net	1950	Video portal
faekalienkanal.com	1928	Video portal
xxxdan.com	1922	Video portal
schnallensex.de	1905	Casual dating
youramateurporn.com	1884	Video portal
tubepornclassic.com	1846	Video portal
picpost.io	1827	Photo portal
hotnudegirls.net	1820	Photo portal
pornq.com	1809	Video portal
wearehairy.com	1806	Video portal
gayfreude.com	1798	Video portal
maturealbum.com	1778	Video portal
bravoporn.com	1742	Video portal
yuvutu.com	1734	Video portal
redwap.me	1722	Video portal
iamxxx.com	1713	Wrongly assigned
vikiporn.com	1683	Video portal
zeige-deine-sexbilder.com	1667	Producers website
xvidzz.com	1662	Video portal
dirtypornvids.com	1657	Video portal
sleazyneasy.com	1651	Video portal
madchensex.com	1642	Video portal
brazzers.com	1624	Video portal
mom50.com	1620	Photo portal
sexstories.com	1618	Eroticism
bilatinmen.com	1614	Video portal
foxgay.com	1607	Video portal

hot-sex-tube.com	1602	Video portal
instasext.com	1594	Casual dating
allover30.com	1583	Prostitution
penis-bilder.com	1550	Photo portal
abenteuerx.com	1537	Casual dating
homemoviestube.com	1530	Video portal
payserve.com	1521	Distributor
fundorado.de	1513	Webcam portal
anysex.com	1507	Video portal
hairyerotica.com	1498	Photo portal
literotica.com	1497	Eroticism
guysinsweatpants.com	1486	Producers website
chatti.de	1482	Eroticism
pornocbs.com	1477	Video portal
eroticbeauties.net	1467	Distributor
nurxxx.net	1465	Video portal
zbporn.com	1464	Video portal
stream-mydirtyhobby.co	1453	Casual dating
fantasti.cc	1441	Producers website
chaturbieren.net	1431	Webcam portal
nursexfilme.com	1404	Video portal
wildesporno.com	1393	Video portal
dirtyroulette.com	1386	Webcam portal
vivud.com	1380	Video portal
bestandfree.com	1378	Video portal
itinyteens.com	1374	Photo portal
shemaleporn.xxx	1355	Video portal
mylust.com	1348	Video portal
theporndude.com	1340	Distributor
asexstories.com	1335	Eroticism
milfgalleries.com	1331	Photo portal
lxax.com	1323	Video portal
camdorado.com	1268	Webcam portal
erogeschichten.com	1259	Eroticism
gaytorrent.ru	1242	Torrent
katestube.com	1241	Video portal
pornwhite.com	1235	Video portal
meteopool.org	1234	Wrongly assigned
ftopx.com	1232	Photo portal
perfectgirls.net	1220	Video portal
gaymenring.com	1219	Video portal
bunnylust.com	1198	Photo portal
dinotube.com	1196	Video portal
pornoxo.com	1185	Video portal
menhdv.com	1182	Video portal
wadtube.com	1180	Video portal
pornovideoshub.com	1179	Distributor
watchmygf.me	1176	Video portal
efukt.com	1163	Eroticism
parispornmovies.com	1163	Video portal
alphaporno.com	1159	Video portal
pornjam.com	1153	Video portal
bigboobsfilm.com	1147	Video portal
mannfuermann.com	1147	Eroticism
machogaytube.com	1136	Video portal
babesjoy.com	1134	Photo portal
erotikum.de	1134	Prostitution
porngem.com	1125	Video portal
livejasminbabes.net	1121	Distributor
gratisxhamster.com	1119	Wrongly assigned
pornoraum.com	1118	Video portal
3dpornpics.pro	1113	Photo portal
inaporn.com	1106	Video portal
azgals.com	1102	Photo portal
porno365.sex	1098	Video portal
pervclips.com	1096	Video portal
tacamateurs.com	1095	Distributor

3movs.com	1094	Video portal
pornopunkt.com	1089	Video portal
pornglee.com	1085	Video portal
candidgirls.io	1084	Video portal
lesarion.com	1074	Wrongly assigned
sexuria.to	1072	Torrent
bestfreetube.xxx	1067	Video portal
besthugecocks.com	1065	Video portal
hd-sexfilme.com	1057	Video portal
pornogrund.com	1045	Video portal
pornomovies.com	1041	Video portal
sexy-legwear.com	1041	Photo portal
forpornstarlovers.com	1037	Wrongly assigned
mangaporno.pro	1033	Erotic cartoons or hentai portal
youngheaven.com	1021	Photo portal
sexygirlspics.com	1014	Photo portal
tubemissile.com	1006	Video portal
goteenpics.com	1003	Photo portal
